# Comparative Risk of Type 2 Diabetes Mellitus Among Vegetarians and Non-Vegetarians

**DOI:** 10.4103/0970-0218.69285

**Published:** 2010-07

**Authors:** Gaffar Sarwar Zaman, Forhad Akhtar Zaman, Mohammad Arifullah

**Affiliations:** Department of Biochemistry, College of Medicine, King Khaled University, Abha, Saudi Arabia. E-mail: drgszaman@gmail.com; 1 Department of Community Medicine, Khaja Banda Nawaz Institute of Medical Sciences, Gulbarga, India; 2 Department of Pathology, Al-Ameen Medical College and Hospital, Bijapur, Karnataka, India

Sir,

The prevalence of type 2 diabetes in vegetarians was compared to that in non-vegetarians in 724 people in the Bijapur district of Karnataka in a hospital-based survey. The type of vegetarian diet was categorized based on a food-frequency questionnaire. A vegetarian diet, in the broad sense, is defined as the one that does not include meat, fish, or fowl. An ovolactovegetarian is a vegetarian who does not eat beef, lamb, pork, poultry, fish, shellfish, or animal flesh of any kind, but is willing to consume dairy and egg products. Pescetarianism, also called pescovegetarianism, is the practice of a diet that includes seafood and excludes mammals and birds. In addition to fish or shellfish, a pescetarian diet typically includes some or all of vegetables, fruit, nuts, grains, beans, eggs, and dairy. Vegetarian diets offer a number of nutritional benefits including lower levels of saturated fat, cholesterol, and animal protein as well as higher levels of carbohydrates, fiber, magnesium, potassium, folate, antioxidants such as vitamins C and E, and phytochemicals.(1)Vegans tend to be thinner, have lower serum cholesterol, and lower blood pressure, reducing their risk of heart diseases and diabetes mellitus. However, eliminating all animal products from the diet increases the risk of certain nutritional deficiencies. Micronutrients of special concern for the vegans include vitamins B_12_and D, calcium, and long-chain *n*-3 (omega-3) fatty acids.(2)On the other hand, the omission of meat and fish from the diet increases the risk of certain nutritional deficiencies.(3)There is convincing evidence that vegetarians have lower rates of coronary heart disease, largely explained by low LDL cholesterol, probable lower rates of hypertension and diabetes mellitus, and lower prevalence of obesity.(4)In our study, mean BMI was lowest in vegans (23.9 kg/m^2^) and incrementally higher in ovolactovegetarians (25.9 kg/m^2^), pescovegetarians (26.4 kg/m^2^), semivegetarians (27.36 kg/m^2^), and non-vegetarians (29.2 kg/m^2^). The prevalence of type 2 diabetes increased from 3.1% in vegans to 8.2% in non-vegetarians; the prevalence was intermediate in ovolactovegetarians (3.6%), pescovegetarians (4.9%), or semi-vegetarians (6.4%). After adjustment for age, sex, ethnicity, education, income, physical activity, television watching, sleep habits, alcohol, and BMI, vegans (OR 0.53 [95% CI 0.36–0.60]), ovolactovegetarians (0.55 [0.47–0.64]), pescovegetarians (0.68 [0.61–0.78]), and semi-vegetarians (0.79 [0.64–0.88]) had a lower risk of type 2 diabetes than non-vegetarians. The results show that increased conformity to vegetarian diets protected against risk of type 2 diabetes.
